# PPE needs in the United States during the COVID‐19 pandemic: An analysis using the GetUsPPE online platform

**DOI:** 10.1002/puh2.65

**Published:** 2023-02-15

**Authors:** Matthew Rubashkin, Taylor Purzycki, Kelsey Coolahan, Charlotte Lee, Daniel J Lurie, Benjamin Batorsky, Alexander Chen, Joanna Calderón, Shuhan He

**Affiliations:** ^1^ Data Department GetUsPPE Washington DC USA; ^2^ Data and ML Engineering Cerebral San Francisco California USA; ^3^ St. George's University True Blue Grenada; ^4^ Emergency Medicine St. Joseph's University Medical Center Paterson New Jersey USA; ^5^ OB/GYN, Tufts University Medical Center Boston Massachusetts USA; ^6^ Department of Psychology University of California Berkeley California USA; ^7^ Ciox Health Alpharetta Georgia USA; ^8^ Yale University New Haven Connecticut USA; ^9^ Department of Community Health Sciences University of California Los Angeles California USA; ^10^ Lab of Computer Science, Department of Emergency Medicine Massachusetts General Hospital Boston Massachusetts USA

**Keywords:** COVID‐19, PPE, public health response

## Abstract

The United States is heavily impacted by the COVID‐19 pandemic starting in 2020. Demand for personal protective equipment (PPE) domestically, along with global surge in demand for PPE during the pandemic, overwhelmed supply chains, leading to acute PPE shortages. This article analyzes the PPE supply and demand in the United States by employing data collected by GetUsPPE, a data hub used throughout the pandemic to coordinate support efforts, including connecting facilities in need of PPE with donated supplies. In this article, PPE requests were examined by facility type (acute vs. non‐acute care), geographic location, and PPE type. The research team observed that PPE demand was dispersed across the United States. In the beginning of the pandemic when demand was highest, most requesting facilities self‐reported as acute care facilities, whereas non‐acute care facilities predominated after June 2020. Additionally, the demand for respirators, disinfecting wipes, gowns, face shields, and surgical masks peaked in response to the first, second, and third waves of COVID‐19. This analysis can be utilized in the future to optimize the tracking of PPE shortages and relief efforts.

## INTRODUCTION

An unprecedented global surge in demand for personal protective equipment (PPE) during the COVID‐19 pandemic overwhelmed existing supply chains [1]. It is likely that four main factors contributed to the US shortage of PPE in 2020. The first factor was a dysfunctional budgeting model in hospital operating systems. Hospitals were encouraged to minimize costs rather than maintain adequate PPE. Next, a significant demand shock was brought on by healthcare system needs as well as panicked citizens‐depleted PPE stockpiles. Third, the federal government did not distribute domestic inventories. Lastly, PPE exported to the United States from other countries was drastically reduced [2].

**FIGURE 1:**
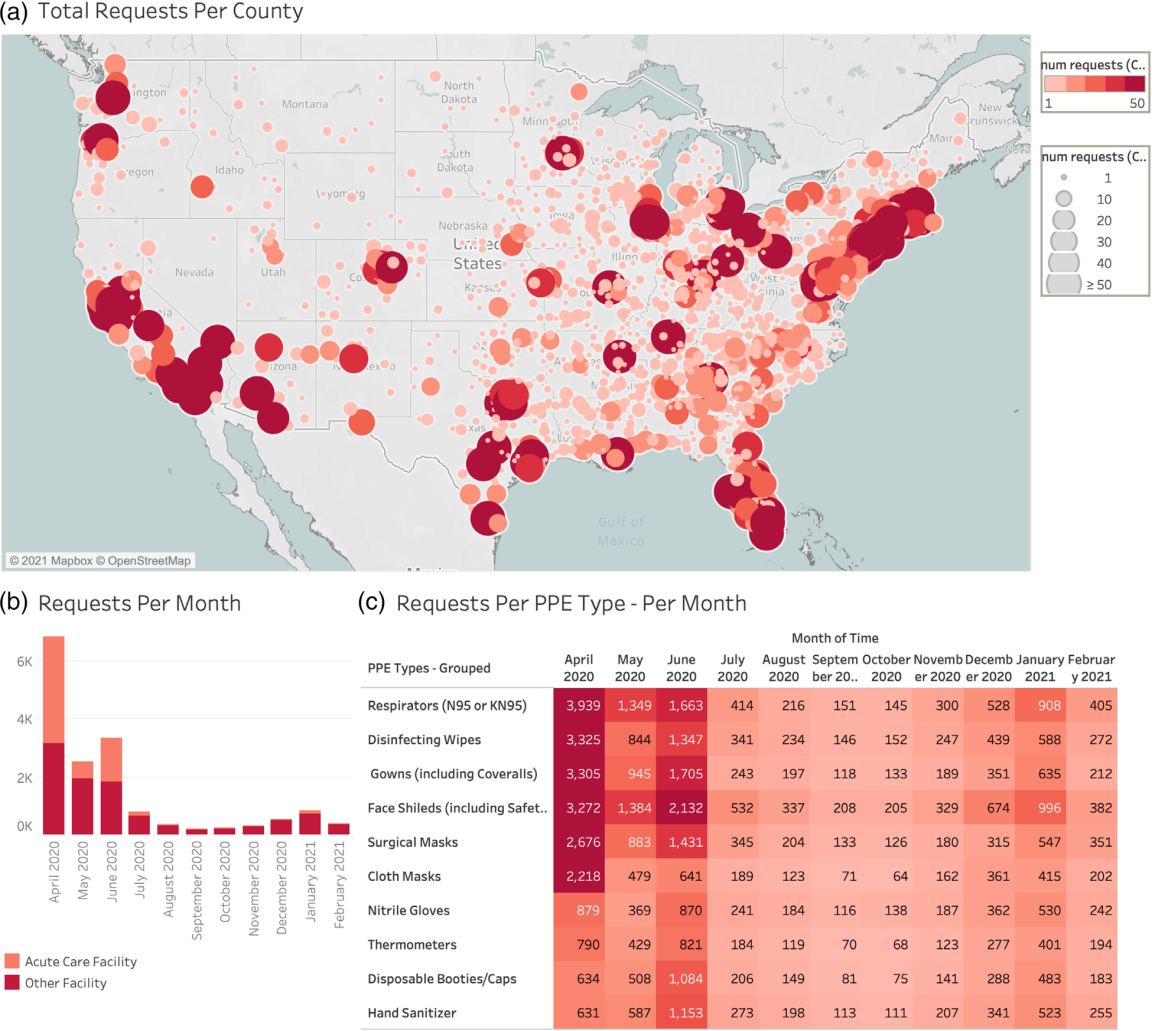
PPE Demand Dashboard indicating Total PPE Requests per County, Total PPE Requests Per Month and Total Requests Per PPE Type. (a) Total PPE Requests Per County, April 2020 to March 2021. Each circle represents a county from which one or more requests were made, sized (1 to 50+) and colored by the number of requests. The greatest densities of requests are clustered in major metropolitan areas, although many counties outside of major cities do have requesting organizations. Data is presented as aggregates over the specified time period. (b) Number of PPE requests per month by facility type. Acute care facilities include organizations that self‐reported as either acute care hospitals, emergency medical services, or other acute care facilities. (c) Number of requests for each type of PPE per month. Facilities could request 0, 1, or more types of PPE. Requests per PPE type are colored by number.

To combat the dwindling supplies, healthcare providers initiated the #GetMePPE movement on Twitter to spread the awareness of the shortage of PPE pandemic, urging public officials to address the PPE shortage. The GetUsPPE.org website was launched to develop a digital platform for the donation, request, and distribution of multimodal sources of PPE. In the United States, GetUsPPE's database was developed to connect facilities in need of PPE with donated supplies [3]. To our knowledge, this is the largest publicly available, longitudinal PPE supply and demand dataset collected in the United States during the COVID‐19 pandemic. We are publishing this article to share our methodology describing in detail the unique PPE demand data and analysis that may help actions in future pandemics (Figure 1). The GetUsPPE platform was initially launched in March 2020 after multiple datasets from other grassroots platforms, including FindTheMasks.org, had been merged. Since that time, data collection has occurred in various forms, including surveys, phone‐banking efforts, and directly through the GetUsPPE website.

GetUsPPE created the GetUsPPE Gateway Application to manage and report on the current state of PPE demand in the United States. The Gateway application was designed and developed in‐house to match the specific task of distributing a scarce resource during crisis response. Gateway uses cloud‐based service Google BigQuery as the database, as well as Heroku, AWS, Redash, and SendGrid to support day‐to‐day operations while also keeping supply and demand data up to date. The data in Gateway includes inbound requests as well as outbound information from facilities requesting PPE.

## TIMELINE OF PPE REQUEST PLATFORM

**March–May 2020**. GetUsPPE platform started with data from the FindTheMasks web platform from March to May 2020. The data collected was incorporated into this analysis. A FindTheMasks Google Form recorded inbound information, including requestor information (name, organization type, address, and recipient role in supply chain) as well as PPE needs (types and condition of acceptance) through an intake form. Public datasets were then used to determine each organization's region, rural–urban status, and county‐level data (including the number of deaths from COVID‐19 for the county the requesting organization was in).

**March–June 2020**. Phone‐banking initiatives were performed by 146+ volunteers in March and June to collect longitudinal, updated data from thousands of facilities that had previously made requests through the GetUsPPE website. Volunteers were assigned facilities to contact and provided scripts to read in an effort to update PPE request information (including supply‐chain contact person, delivery instructions, and updated PPE type and quantity requested). In‐between phone banks, emails were sent to supply‐chain representatives inviting updated PPE requests to ensure that donations were directed to facilities currently in the greatest need.

**April–May 2020**. Informed by the experiences of on‐the‐ground regional organizing teams (e.g., GetMePPE Bay Area and Chicago) who coordinated and facilitated local donations during the initial surge, GetUsPPE developed an expanded request form that collected significant additional information regarding facility characteristics (e.g., services provided and populations served) and PPE needs (e.g., supply remaining for each type of PPE, PPE conservation, and reused practices). This expanded request form (setup on the Survey Monkey platform) was initially sent via email to all facilities who had requested PPE to the FindTheMasks survey, and the expanded request form was subsequently put into use on the GetUsPPE website as part of a custom‐built web‐based request system that began operating in early June.

**May–June 2020**. In May and June 2020, GetUsPPE conducted follow‐up outreach efforts to (1) update historical PPE requests, (2) complete any missing facility and/or contact information, and (3) collect new variables introduced for the equitable distribution of PPE (e.g., PPE use and conservation practices, populations served). This effort was conducted through automated emails followed by coordinated phone‐banking. In late May, all requesting entities who provided a valid email address were sent an automated email. This email asked recipients to update their request and contained a custom URL which pre‐populated the form with data provided in their previous request. In June, GetUsPPE initiated a 3‐week phone banking effort to call requestors who had not responded to the email or for whom a valid email address was not on file. Facilities were given the option to complete the update over the phone or virtually through the custom URL.

**June 2020–March 2021**. In order to ensure that facilities and organizations that serve high‐risk communities are represented in the database, GetUsPPE continued to lead proactive outreach through the Healthcare Outreach branch. This team conducted outreach to facilities and organizations that are at high risk of COVID‐19 infection, hospitalization, and death and encourages them to complete our request form if they are in need of PPE. The two‐fold approach entails identifying high‐risk facilities and populations using CDC guidance and geographic targeting. Geographic targeting takes into account risk factors specific to each facility/population, community‐level risk factors (COVID Community Vulnerability Index), and COVID‐19 forecasting models (Covid Act Now). These outreach efforts help capture the needs of historically and currently underserved communities and allow for more equitable allocation of PPE.

## REVIEW OF THE PPE REQUESTS

The GetUsPPE Gateway BigQuery database was queried in March of 2021 to obtain data from March 2020 till February 2021. Queries were conducted in SQL and are open‐sourced and available on GetUsPPE's GitHub repo. Data was further processed and visualized in Tableau Desktop version 2020.2. Both aggregated and anonymized to county‐level data requests can be found at https://github.com/GetUsPPE/ppe_needs_retrospective/tree/main/data. Anonymized data include the timestamp, state, county, FIPS, as well as facility type(s), and PPE type(s) requested if available. Additionally, our raw SQL scripts used to pull the aggregate data can be found in the GitHub repo.

Numerical PPE request data from mid March 2020 to mid July 2020 was converted to binary yes/no data based on whether or not each type of PPE was included for each request. After slicing the United States PPE request data by location (US state), we then calculated the percentage of requests in each time period (days, weeks, and months) that requested each type of PPE relative to all requests (inclusive of all types of PPE) made during that same epoch. As there were large differences in the number of requests received at different points in time and from different regions, this process was conducted in order to better assess and illustrate the trends in changing relative needs for different PPE types over time and geographic areas, as opposed to the absolute needs for different PPE types.

Data from March–June 2020 was analyzed for the evidence of duplicate requests. We defined a duplicate as an inbound request from the same requestor, for the same facility (as defined by address). We excluded from this analysis requests that originated from the GetUsPPE outbound initiated workflows—that is, distinct follow‐up requests with new PPE needs. Under this criterion, we found that a small fraction of the dataset contained potential duplicates. Specifically, ∼1.2% of requests were identified as duplicates.

Request data collected between April and October 2020 was evaluated for the accuracy of self‐reported facility type: acute or non‐acute care. Acute care facilities included hospitals, field hospitals, hospital overflow facilities, emergency medical services, urgent care clinics, and freestanding emergency departments. Non‐acute care facilities included outpatient medical and dental clinics, nursing homes, schools, homeless shelters, and other essential nonhospital facilities. To assess the reliability of this primarily self‐reported categorization, a random subset of requests made in each month from April to October 2020 were randomly selected and manually verified, through address confirmation and online investigation. This investigation verified the facility‐type trends observed in the self‐reported data. Data verification was performed for all entries in June, confirming high consistency in the trends specifically after the implementation of the expanded intake request form.

## AN ANALYSIS OF THE PPE DATA

Tracking unmet PPE demand is necessary to triage distribution to facilities in greatest need and to guide policy [4, 5]. It is imperative to analyze large‐scale data‐driven distribution of PPE donations GetUsPPE collected, supply remaining, and demand data from facilities since March 2020 to facilitate and guide triage actions. The following are the findings published online in May 2020 collected through March 2021 about PPE needs, PPE supply remaining, and facility characteristics [3]. The data is presented as aggregates over specified timescales. Anonymized cross‐sectional data is available for researchers in the GitHub repository.

GetUsPPE received more than 21,000 requests from organizations and individuals in need of PPE over the first year of the pandemic year. We analyzed how PPE needs and supply levels evolved over the year. The greatest number of requests originated from states with the highest number of cases, including California, New York, Massachusetts, and Florida^5^ (Figure 1). Historically, pandemics have tended to hit big cities the hardest. California, the most populous state in the United States, had the highest number of cases throughout the pandemic. New York and Florida, the third and fourth most populous states respectively, also followed this pattern and were in the top 5 states with the most cases throughout the pandemic. States with larger populations require more medical facilities. Due to the greater number of facilities, more requests were placed from these states. Massachusetts has a comparatively smaller population; however, when looking at population density, it is one of the most densely populated states. Densely populated states are more prone to infectious diseases due to a higher chance of infection in crowded environments.

**FIGURE 2:**
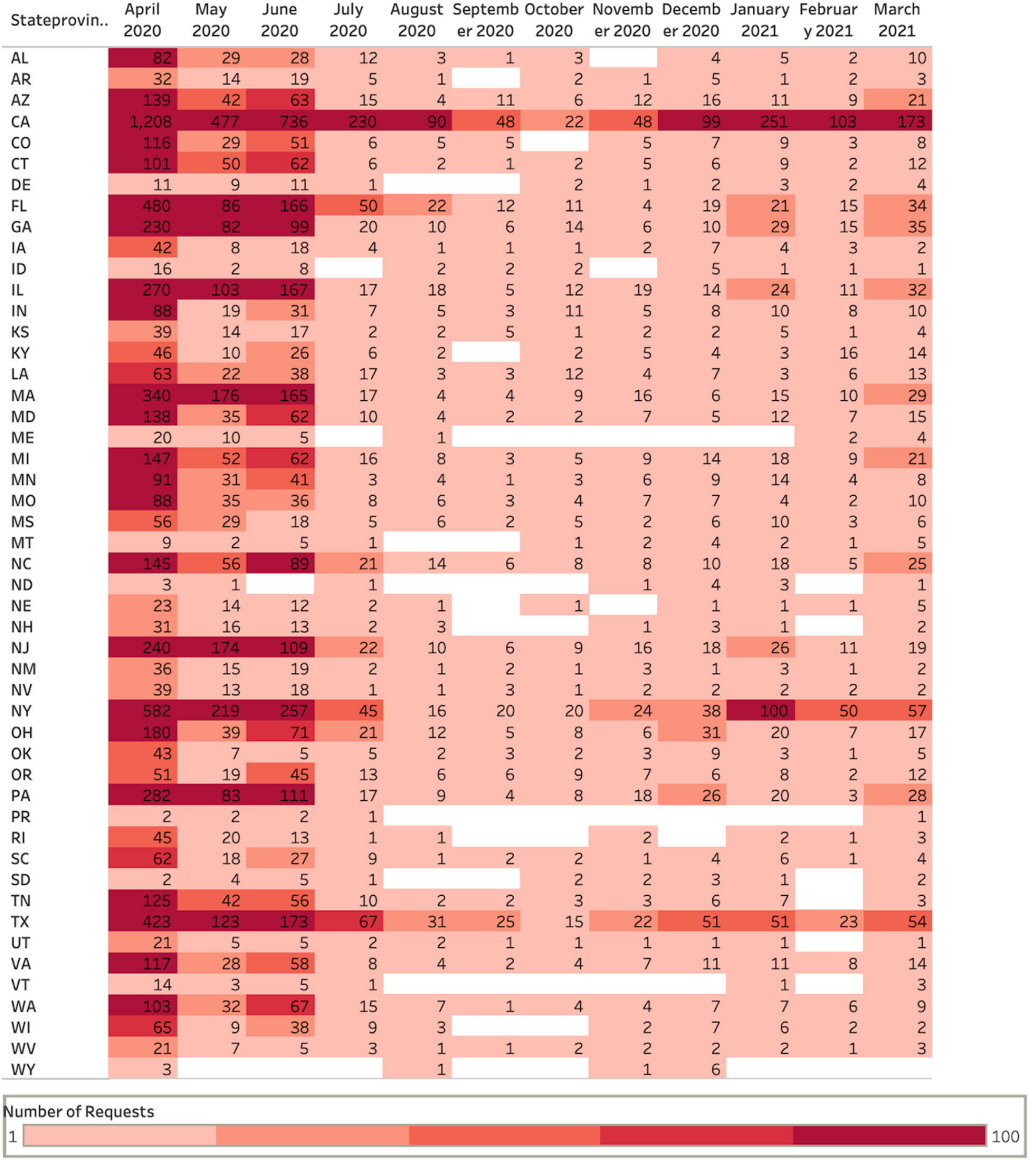
PPE Requests by State and Time. Number of overall requests per state and per month. Each entry represents a single‐PPE request independent of the type or number of different PPEs requested, from April 2020 to March 2021. The greatest density of requesting organizations per state is clustered in states with major urban areas.

Overcrowding housing, a common finding in high‐density populations, is a breeding ground for infectious diseases [6]. These inevitable living conditions made social distancing practices challenging. Urban and coastal regions accounted for the majority of observed demand. Each circle represents a county from which one or more requests were made, sized (1 to 50+) and colored by the number of requests. The greatest densities of requests are clustered in major metropolitan areas, although many counties outside of major cities do have requesting organizations. Data are presented as aggregates over the specified time period. Figure 2 shows each entry represents a single PPE request independent of the type or number of different PPE requested, from April 2020 to March 2021. The greatest density of requesting organizations per state is clustered in states with major urban areas. Big cities and coastal regions are hubs for international transport networks, allowing infection to easily spread city to city. Infection rates were subsequently higher and led to the population seeking medical attention. Therefore, facilities from these regions accounted for the majority of observed demand.

Demand for PPE peaked in April 2020, with demand resurging in June 2020 and January 2021. In March 2020, the WHO declared COVID a pandemic. One month later (April 2020), the CDC recommended that all people wear a mask when outside of the home, causing a significant increase in the amount of PPE requests [7]. Acute care facilities such as hospitals, emergency response services, and urgent care facilities comprised 55% of requests in April 2020. In May 2020, non‐acute care facilities such as nursing homes, outpatient medical and dental clinics, and schools comprised 75% of PPE requests.

**FIGURE 3:**
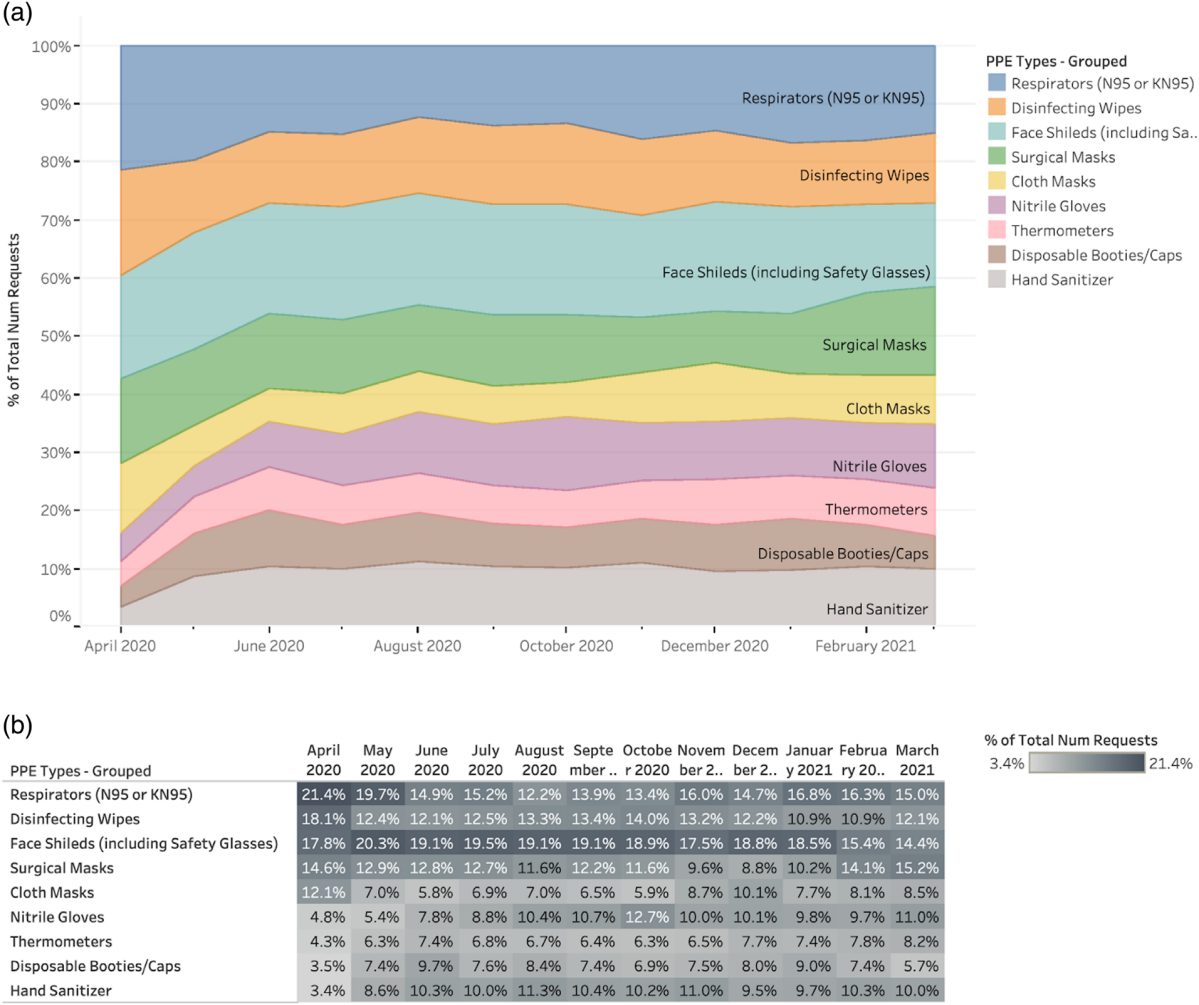
Comparative percentage of PPE requests per month, including specific PPE type. (a) Chart of normalized requested amounts of PPE types, calculated by the total amount of specific PPE type requests per month. The data indicates that N95 respirators, disinfecting wipes, gowns, face shields, and surgical masks were in high demand throughout the crisis. (b) Table of normalized requested amounts of PPE types, calculated by the total amount of specific PPE type requests per month. The table is shaded by percent of total number of requests from light (3.4%) to dark (21.4%). The data used in this table can be used to easily comparing changes across months. We observed that across all PPE types, the number of requests varied by month but the normalized need remained relatively consistent.

Non‐acute care facilities comprised nearly all incoming requests (93%) as of March 2021 (Figure 3). Facilities could request 0, 1, or more types of PPE. Requests per PPE type are colored by number. Acute care facilities include organizations that self‐reported as either acute care hospitals, emergency medical services, or other acute care facilities. Cases increased due to relaxed restrictions, prompting more PPE requests. With return to normal life, PPE was especially needed in non‐acute care facilities versus acute care facilities. A rise was also seen in January 2021, likely due to holiday gatherings and travel [8]. Furthermore, delta and omicron began to circulate at the same time [9].

N95 respirators, disinfecting wipes, gowns, face shields, and surgical masks were in high demand throughout the crisis. Numerical PPE request data was converted to binary yes/no data based on whether or not each type of PPE was included for each request. We then calculated the percentage of requests in each time period (month) that requested each type of PPE relative to all requests (inclusive of all types of PPE) made during that same time period. This process was conducted to better assess and illustrate the trends in changing relative needs for different PPE types over time, as opposed to the absolute needs for different PPE types. The number of requests varied by month but the normalized need remained relatively consistent (Figure 3).

Diving deeper, we analyzed PPE requests reporting No Supply Remaining of specific PPE types, per month. PPE supply remaining data was converted to categorical values of 0 supply remaining, 1–7 days remaining or 7+ days remaining—then normalized to total requests in the analyzed time frame. If an inbound request did not include a specific PPE type, or no supply remaining information was provided, that data was not included in the analysis. This process was conducted to better assess and illustrate the trends in changing relative supply of different PPE types over time, and to highlight PPE requestors reporting no supply remaining.

**FIGURE 4:**
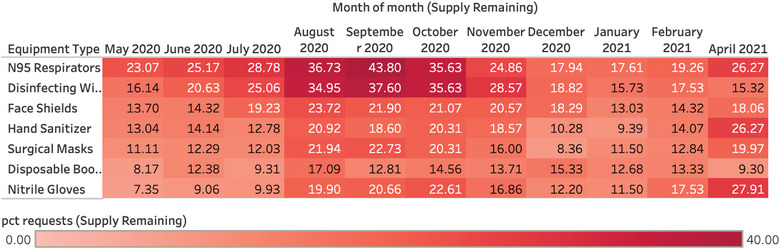
Reported No Supply Remaining of Specific PPE Types. PPE requests reporting No Supply Remaining of specific PPE types, per month. PPE supply remaining data was converted to categorical values of 0 supply remaining, 1–7 days remaining or 7+ days remaining—then normalized to total requests in the analyzed time frame. The most acute shortages of PPE supply remaining occurred in the summer months of 2020 when COVID cases were at lower levels across the United States.

Notably, supply of PPE remaining varied by PPE type and over time. N95 respirators had the most acute shortages, with 0 supply remaining reported by 17%–40% of respondents, depending on time period (Figure 4). The most acute shortages of PPE supply remaining occurred in the summer months of 2020 when COVID cases were at lower levels across the United States during the first year of the pandemic. We hypothesize that we may have oversampled institutions with more acute supply shortages, as COVID cases were decreasing across the United States, and we received the least amount of total PPE requests in this time period.

Important to note are the limitations of the methodology used. All data are self‐initiated and self‐reported PPE needs of individuals and facilities in need at the time of data capture. As such, the data is not necessarily a representative sample of PPE needs across time, geography, or type of facility. The fluctuation of requests over time and geographical location may also be driven in part by changes in awareness of GetUsPPE as an organization.

## CONCLUSIONS FOR THE FUTURE

Trends indicate an evolving need for PPE that coincided with increasing COVID‐19 cases in April 2020, June 2020, and January 2021 in the United States^5^ [10]. At the end of the first year of the pandemic, non‐acute facilities continued to face acute PPE shortages, as compared to trends seen across most acute facilities. We hypothesize that large hospital systems benefited first from a slowly recovering PPE supply chain.

With a recovering and fragile PPE supply chain, data‐driven solutions for tracking PPE demand are highly warranted and serve to identify and improve issues in the PPE supply chain in future crises. The GetUsPPE methodology and database can be utilized in the future to optimize the tracking of facility PPE shortages and distributions longitudinally. Analyses of need and the efforts to improve awareness, establish trust with the public, and ultimately provide frontline workers with much‐needed protective equipment are ongoing.

## AUTHOR CONTRIBUTIONS

*Conceptualization; data curation; formal analysis; investigation; methodology; project administration; supervision; visualization; writing—original draft; writing—review and editing*: Matthew Rubashkin. *Data curation; writing—original draft; writing—review and editing*: Taylor Purzycki. *Conceptualization; data curation; writing—review and editing*: Kelsey Coolahan. *Conceptualization; investigation; methodology*: Charlotte Lee. *Conceptualization; data curation; methodology; writing—review and editing*: Daniel J Lurie. *Data curation; formal analysis; methodology*: Benjamin Batorsky. *Data curation; formal analysis*: Alexander Chen. *Conceptualization; investigation; methodology; project administration; supervision; writing—original draft*: Shuhan He.

## CONFLICT OF INTERESTS STATEMENT

The authors declare that there is no conflict of interest that could be perceived as prejudicing the impartiality of the research reported.

## Data Availability

The data that support the findings of this study are openly available in GitHub at https://github.com/GetUsPPE/ppe_needs_retrospective
